# The Children of the State

**Published:** 1896-06-06

**Authors:** 


### Modern Sociology.

### THE CHILDREN OF THE STATE.

It is an axiom with some that the existence of pauperism is a sign of failure in the social system; and the evolution of a state of society of which paupers shall form no part is the dream of no small number of social reformers. Nevertheless, in practical everyday life the pauper is a fact which cannot be ignored. In England he has his legal rights, but even in countries where the pauper has no such rights he exists just the same as anywhere else, and, in one form or another, provision has to be made for him. Everywhere, then, it becomes a matter of the profoundest interest that his existence should be discouraged, and his numbers be kept down at the lowest possible figure, for wherever he is found the non-worker is a parasite living on the labour of the working portion of the community.

The whole mechanism of the Poor Law is founded on the intention of making pauperism unattractive, giving the necessaries of life, but giving them in such a manner as to deter all but the absolutely destitute from asking for them. The one excuse for what we may call the barbarism of the Poor Law, the one thing which reconciles guardians possessing an average share of humanity to the harsh treatment of the poor, and even of the sick and aged, which is common in some workhouses, is that it is deterrent and that some form of determent is considered absolutely necessary.

What, then, are we to think of that other side of the Poor Law administration which, under the pretence of educating pauper children, actually manufactures paupers?

Whatever else we may gather from the report of the Committee on Pauper Schools which was issued some time ago, this much is clear enough, that instead of turning out bright, intelligent, honest young people, ready and anxious to make their way in the world, these schools turn out, year after year, a crowd of poorly developed children, ignorant to a degree of the ways of the world into which they are thrust forth; ready to steal, not exactly from dishonesty, but because never having possessed anything they do not appreciate the meaning of possession; untrained in any but the roughest sorts of work; unfitted for any calling, except to fill the already overcrowded ranks of unskilled labour; but fully trained, from their birth upwards, to dependency on others, and with their minds ingrained with that "institution" helplessness which, when they attempt to earn their living, leaves them strangers in a strange land, until they drift again into the ranks of rate-supported pauperism, and, after begetting a few children to become paupers like themselves, they end their career where they began it—at the workhouse door. Surely if the Poor Law is to be repressive it should be repressive from the very beginning and should take care that, however much the fancies and idiosyncrasies of parents may spoil the bringing up of the children of freedom, the children of the State at least should show what can be done by education in the production of good citizens.

The first and most important duty of the State in the education of the children who may be thrown upon its care is to separate them in every way from all possible taint or suggestion of pauperism. They should not be workhouse children, they should grow up alongside of the ordinary children of the country, and under no circumstances should they be allowed to become a class apart. The very fact that the children in the great barrack schools are of necessity a class apart is enough to condemn such institutions, quite irrespective of the other evils connected with them which were described to the committee which recently inquired into the subject.

The bringing up of children is the special work and special duty of the Education Department, and it seems most right and reasonable that, when the State has children thrown upon its hands, that department should undertake their control and education; especially in view of the recognised necessity of separating them from all contact with the mechanism, and even with the officers of the Poor Law. We are, therefore, fully in accord with the clause in the new Education Act allowing such a transfer of duty from the guardians to the education authorities, and even more fully in accord with the deputation which lately waited on the Duke of Devonshire, the Lord President of the Council, to urge that this clause should be strengthened in the direction of making it obligatory instead of optional.

At the same time, it would be foolish to shut one's eyes to the difficulties with which the question is surrounded. If it were the fact that when a parent was once a pauper he was always a pauper, and that when once his children had been handed over to the authorities the State was free to deal with them in such a manner as might best conduce to the making of good citizens, there could be but little doubt that a universal system of "boarding out" would be the best plan to adopt—a plan by which these erratic wastrels might be grafted upon the good stock of the steady working population, and restored again to the position they would have held but for the errors or misfortunes of their parents.

The matter is immensely complicated, however, by the rights of parents and by the large strain of mental imperfection and bodily weakness which pervades the children.

There are hundreds, nay thousands, of people who use the workhouses as temporary homes, flying to them during the cold months, or when times are bad, and there are many more who, either from defective health or deficient will, can only earn their living in an intermitting fashion, living in workhouses in the intervals. Now, as things are at present, this great tribe of "ins and outs," who oscillate between workhouse life and low-class freedom, drag with them a still greater tribe of children, who are, by these frequent changes, debarred from all continuity of education, and play havoc with the education of those with whom they mix when they return to the guardianship of the Poor Law. Nor must it be forgotten that both chronic pauperism and criminality are associated with forms of degeneracy which are more or less hereditary, and that in the very nature of things, children of such an ancestry are often incapable of benefiting by the sort of education which is found most suitable for normal children. It is clear, then, that there will be considerable difficulties in entirely abolishing large schools.

Children who are imperfect and peculiar can only be made the most of by a very special kind of training, which is hardly possible except in large institutions, while, unless we are prepared to modify considerably the ideas now current as to the rights and responsibilities of parents, the "ins and outs," the children of intermitting paupers, will be a constant difficulty.

